# Morphological Regeneration and Functional Recovery of Neuromuscular Junctions after Tourniquet-Induced Injuries in Mouse Hindlimb

**DOI:** 10.3389/fphys.2017.00207

**Published:** 2017-04-06

**Authors:** Huiyin Tu, Dongze Zhang, Ryan M. Corrick, Robert L. Muelleman, Michael C. Wadman, Yu-Long Li

**Affiliations:** ^1^Department of Emergency Medicine, University of Nebraska Medical CenterOmaha, NE, USA; ^2^Department of Cellular and Integrative Physiology, Nebraska Medical CenterOmaha, NE, USA

**Keywords:** injury, ischemia-reperfusion, motor nerve terminal, neuromuscular junction, nicotinic acetylcholine receptor, tourniquet

## Abstract

Tourniquet application and its subsequent release cause serious injuries to the skeletal muscle, nerve, and neuromuscular junction (NMJ) due to mechanical compression and ischemia-reperfusion (IR). Monitoring structural and functional repair of the NMJ, nerve, and skeletal muscle after tourniquet-induced injuries is beneficial in exploring potential cellular and molecular mechanisms responsible for tourniquet-induced injuries, and for establishing effective therapeutic interventions. Here, we observed long-term morphological and functional changes of the NMJ in a murine model of tourniquet-induced hindlimb injuries. Unilateral hindlimbs of C57/BL6 mice were subjected to 3 h of tourniquet by placing an orthodontic rubber band, followed by varied periods of tourniquet release (1 day, 3 days, 1 week, 2 weeks, 4 weeks, and 6 weeks). NMJ morphology in the gastrocnemius muscle was imaged, and the endplate potential (EPP) was recorded to evaluate NMJ function. In NMJs, nicotinic acetylcholine receptor (nAChR) clusters normally displayed an intact, pretzel-like shape, and all nAChR clusters were innervated (100%) by motor nerve terminals. During 3 h of tourniquet application and varied periods of tourniquet release, NMJs in the gastrocnemius muscle were characterized by morphological and functional changes. At 1 day and 3 days of tourniquet release, nAChR clusters retained normal, pretzel-like shapes, whereas motor nerve terminals were completely destroyed and no EPPs recorded. From 1 to 6 weeks of tourniquet release, motor nerve terminals gradually regenerated, even reaching that seen in sham mice, whereas nAChR clusters were gradually fragmented with prolongation of tourniquet release. Additionally, the amplitude of EPPs gradually increased with prolongation of tourniquet release. However, even at 6 weeks after tourniquet release, the amplitude of EPPs did not restore to the level seen in sham mice (13.9 ± 1.1 mV, *p* < 0.05 vs. sham mice, 29.8 ± 1.0 mV). The data suggest that tourniquet application and subsequent release impair the structure and function of NMJs. Morphological change in motor nerve terminals is faster than in nAChR clusters in NMJs. Slow restoration of fragmented nAChR clusters possibly dampens neuromuscular transmission during the long phase following tourniquet release.

## Introduction

In vertebrates, the skeletal muscle requires motor nerve innervation to produce skeletal muscle contractions and to avoid muscle atrophy. Contact between motor neurons and their target muscle fibers forms a chemical synapse, known as the neuromuscular junction (NMJ). In the physiological condition, excited motor neurons release acetylcholine (ACh) from their nerve terminals, which diffuses across the synaptic cleft and binds to nicotinic acetylcholine receptors (nAChRs) on the postsynaptic membrane (known as the motor endplate) of the muscle. Binding of ACh to nAChRs can depolarize endplate potential (EPP) and may trigger a cascade of signals that eventually results in skeletal muscle contractions and movements (Tintignac et al., 2015).

Efficient neuromuscular transmission is highly reliable, in that a high density of postsynaptic nAChRs (about 10,000 receptors/μm^2^) is maintained in the motor endplate (Sanes and Lichtman, 2001) and redistributed to form nAChR clusters (Froehner et al., 1990; Phillips et al., 1991; Yu and Hall, 1994; Huh and Fuhrer, 2002). These compacted nAChR clusters are considered as safety mechanism in that the excess depolarization of the postsynaptic membrane in response to each nerve impulse ensures the occurrence of skeletal muscle contractions in healthy tissue (Wood and Slater, 2001).

Proper development and organization at the NMJ are necessary for effective neuromuscular transmission (Tintignac et al., 2015). However, a number of pathological conditions that may affect the innervation and distribution of nAChRs can lead to a reduction in the safety mechanism and result in the impairment of neuromuscular transmission (Wood and Slater, 2001). Injured peripheral nerves and skeletal muscles have a remarkable ability for tissue regeneration (Scheib and Hoke, 2013; Domingues-Faria et al., 2016), and regeneration of functional NMJs occurs after NMJ injury (McMahan et al., 1980; Darabid et al., 2014). Although, regenerated NMJs are mainly formed at original synaptic sites of the skeletal muscle (McMahan et al., 1980), growing evidence demonstrates that NMJs are also formed on regenerated axons and muscle fibers (Slater, 1982; Slater and Allen, 1985; Ijkema-Paassen et al., 2002; Nishizawa et al., 2003; Li and Thompson, 2011; Grumbles et al., 2012; Kang and Lichtman, 2013). The alteration of regenerated NMJs formed at both original synaptic sites and new synaptic areas, however, is associated with permanent neurological deficiency, and long-term skeletal muscle contractile dysfunction, even to the degree of complete limb paralysis (Creager et al., 2012).

The tourniquet is commonly used for severe limb hemorrhage and is often necessary to save life (Beekley et al., 2008; Inaba et al., 2015; Zietlow et al., 2015; Scerbo et al., 2016). However, a number of complications, including serious injuries to the skeletal muscle, nerve, and NMJ, are related to both tourniquet treatment and subsequent tourniquet release (Ochoa et al., 1971; Kam et al., 2001; Doyle and Taillac, 2008). Injuries from tourniquet-induced direct mechanical compression and ischemia-reperfusion (IR) have made recommended use of the tourniquet controversial and ambiguous. Animal models of tourniquet-induced limb injuries have been widely used to mimic human tourniquet-related extremity injuries for studying the pathophysiology and consequences of tourniquet-induced limb injuries. Using a mouse model of tourniquet-induced hindlimb injuries, our previous studies showed that 3 h of tourniquet application and 4 h of subsequent tourniquet release caused necrosis and apoptosis of the gastrocnemius muscle (Tran et al., 2011, 2012). Additionally, after 3 h of tourniquet followed by 6 weeks of tourniquet release, function of the NMJ, and skeletal muscle contractility are only partially recovered (Zhang et al., 2017). Monitoring morphological and functional changes of the NMJ and skeletal muscle after tourniquet application is beneficial to exploring the potential cellular and molecular mechanisms responsible for tourniquet-induced limb injuries, optimizing the tourniquet method to reduce major and direct damage to innervating motor axons and NMJs, and discovering effective therapeutic interventions to improve long-term recovery of the skeletal muscle, nerve, and NMJ from tourniquet-induced injuries. In the present study, we observed the time course for morphological and functional alterations of the NMJ in a murine model of tourniquet application and subsequent release at the hindlimb. We found that tourniquet application and subsequent release significantly altered the morphology of NMJs. In particular, motor nerve terminals and nAChR clusters did not present synchronizing regeneration in NMJs located on gastrocnemius muscles, which affected recovery of the neuromuscular transmission.

## Materials and methods

### Animals

Male C57/BL6 mice (7–8 weeks of age, 22–24 g, *n* = 65, Charles River Laboratory) were housed under controlled temperature and humidity and a 12:12-h dark–light cycle, and were provided water and mouse chow *ad libitum*. Body weights of animals in different time-points of treatments were shown in Table 1. Experiments were approved by the University of Nebraska Medical Center Institutional Animal Care and Use Committee and were carried out in accordance with the National Institutes of Health (NIH Publication No. 85–23, revised 1996) and the American Physiology Society's “Guides for the Care and Use of Laboratory Animals.”

**Table 1:** **Body weight in sham mice and mice with different periods of the tourniquet release after 3 h of tourniquet (1-d TR, 3-d TR, 1-wk TR, 2-wk TR, 4-wk TR, and 6-wk TR)**.

	**Body weight (g)**					
	**1 day**	**3 days**	**1 week**	**2 weeks**	**4 weeks**	**6 weeks**
Sham mice	22.8 ± 0.2 (3)	22.9 ± 0.3 (3)	23.1 ± 0.2 (3)	24.3 ± 0.2 (4)	25.9 ± 0.2 (4)	26.9 ± 0.3 (4)
TR mice	22.2 ± 0.3 (6)	22.6 ± 0.3 (7)	22.8 ± 0.2 (8)	24.0 ± 0.3 (8)	25.5 ± 0.3 (8)	26.7 ± 0.2 (7)

Data are means ± SEM. Numbers in parentheses indicate the number of animals. ^*^P < 0.05 vs. age-matched sham mice.

### A mouse model of tourniquet-induced hindlimb injuries

A mouse model of tourniquet-induced hindlimb injuries has been used in our previous studies (Tran et al., 2011, 2012; Zhang et al., 2017). Mice were anesthetized with a cocktail consisting of 100 mg/kg ketamine and 10 mg/kg xylazine, given as an intraperitoneal injection (0.01 ml/g body weight). The level of anesthesia was continuously monitored by observing the respiratory patterns and toe pinch reflex. Anesthesia was maintained throughout 3 h of tourniquet and initial 4 h of tourniquet release with additional anesthetic cocktail (0.1 ml) as needed. After the induction of anesthesia, fur was completely removed from left hindlimb with an electric shaver. The animals were restrained on a heating pad to maintain body temperature at 37°C until the animals woke up.

The tourniquet in unilateral hindlimb (left) was performed by placing an orthodontic rubber band at the hip joint, using a McGivney hemorrhoidal ligatorer. After 3 h of tourniquet, the orthodontic rubber band was released and the hindlimb underwent tourniquet release for 1 day (1-d TR), 3 days (3-d TR), 1 week (1-wk TR), 2 weeks (2-wk TR), 4 weeks (4-wk TR), or 6 weeks (6-wk TR), respectively. Sham-operated animals were subjected to the same procedure except for the application of the orthodontic rubber band. During 3 h of tourniquet application and the initial 4 h of tourniquet release, mice were kept well-hydrated with an intraperitoneal injection of 0.2 ml normal saline every 2 h.

### Electrophysiological recording of the EPP *in situ* (Zhang et al., 2017)

Under anesthesia (800 mg/kg urethane and 40 mg/kg chloralose, i.p.), the mouse was placed in prone position and maintained on a heating pad at 37°C. The middle and distal end of the left gastrocnemius muscle was isolated and then covered by warmed saline-moistened cotton. The distal cut end of the exposed sciatic nerve was placed on a bipolar platinum electrode and covered by cotton moistened with mineral oil. A specific muscle Na^+^ channel blocker, μ-conotoxin GIIIB (4 μM, 200 μL) was locally injected into the gastrocnemius muscle to inhibit muscle contraction, and muscle contraction was fully blocked 15 min after drug injection. The EPP were recorded by intracellular recording technique. A glass microelectrode filled with 3 M KCl (5–15 MΩ pipette resistance) was slowly inserted into the gastrocnemius muscle fiber and then connected with an intracellular preamplifier (IX 1; Dagan Corporation, Minneapolis, MN, USA). Proximity of the electrode to an endplate was determined by the presence of miniature endplate potentials (mEPPs). Sciatic nerve stimulation (10 V, 50 Hz, 0.1 ms) was used to evoke EPP. Recording of sciatic nerve stimulation-evoked EPPs was digitized and stored on computer by PowerLab 8/30 Data Acquisition System with LabChart 7 (AD Instruments) for analyzing the amplitude of EPPs. EPPs were recorded in 10–15 sites of the gastrocnemius muscle from each mouse in all experimental groups.

### Fixation and dissection of muscle

At the end of sham or tourniquet-to-tourniquet release protocol, the mouse was anesthetized by intraperitoneal injection of ketamine (100 mg/kg) and xylazine (10 mg/kg) solution. The gastrocnemius muscle was immediately harvested and postfixed with 4% paraformaldehyde in 0.1 M phosphate-buffered saline (PBS) for 15 min, followed by incubation in 0.1 M glycine for 15 min. The muscle was split into 8–10 small longitudinal segments to facilitate penetration of probes into neuromuscular junctions.

### Immunohistochemistry of NMJs

Split segments of the gastrocnemius muscle were permeabilized in −20°C methanol for 10 min and blocked for 1 h in PBS containing 0.5% Triton and 1% BSA. Muscle segments were subsequently incubated overnight at 4°C in a cocktail of primary antibodies diluted in blocking solution. Axons and nerve terminals were labeled with mouse anti-neurofilament 200 (Sigma. N0142, 1:500) and rabbit anti-synaptophysin (ThermoFisher. MA5-16402, 1:100) antibodies. After several rinses with PBS, muscle segments were then incubated overnight at 4°C with Alexa Fluor® 594-labeled donkey anti-mouse (invitrogen. A21203, 1:200) and anti-rabbit (invitrogen. A21207, 1:200) IgGs, and Alexa Fluor® 488 labeled α-Bungarotoxin (α-BTX, invitrogen. B13422, 1:100). Muscle segments were thoroughly rinsed with PBS and mounted on glass slides. Immunohistochemically labeled NMJs were imaged using a laser scanning confocal microscope (Zeiss LSM 710).

### Quantification of the NMJ morphology and imaging

Z-stack images of the NMJ were obtained from five different regions of each muscle segment and illustrated with maximum intensity projection. All analyses were performed on en-face NMJs. For quantification of the nAChR area in the NMJ, all endplate sizes labeled with α-BTX were estimated by manual tracing in ImageJ software (NIH Image) to calculate the number of discrete fragments per nAChR cluster, the nAChR area per nAChR cluster, the area per fragment, and the percentage of fragmented nAChR clusters in total nAChR clusters. Nerve terminal sizes labeled with neurofilament and synaptophysin in the endplate were measured to quantify the percentage of nerve innervation and the percentage of nerve terminal occupancy in NMJs. In the NMJ, the endplate with or without labeling for neurofilament and synaptophysin serves as an innervated or denervated endplate. The number of discrete fragments per nAChR cluster ≥5 was considered as a fragmented nAChR cluster. The nerve terminal occupancy in NMJs was calculated by overlapped area of the motor nerve terminal, with the nAChR cluster divided by total area of the nAChR cluster.

### Data analyses

All data are presented as mean ± SE. SigmaPlot 12 (Systat Software, Chicago, IL) was used for data analyses. One-way ANOVA, with a Bonferroni procedure for *post-hoc*, was used to determine statistical significance for multi-group comparison. A Chi-Square test was performed for percentage of fragmented nAChR clusters in total nAChR clusters and percentage of nerve innervation in the NMJ. Statistical significance was accepted when *P* < 0.05.

## Results

### Changes of the NMJ morphology during the tourniquet and subsequent release (TR)

**Figure 1:**
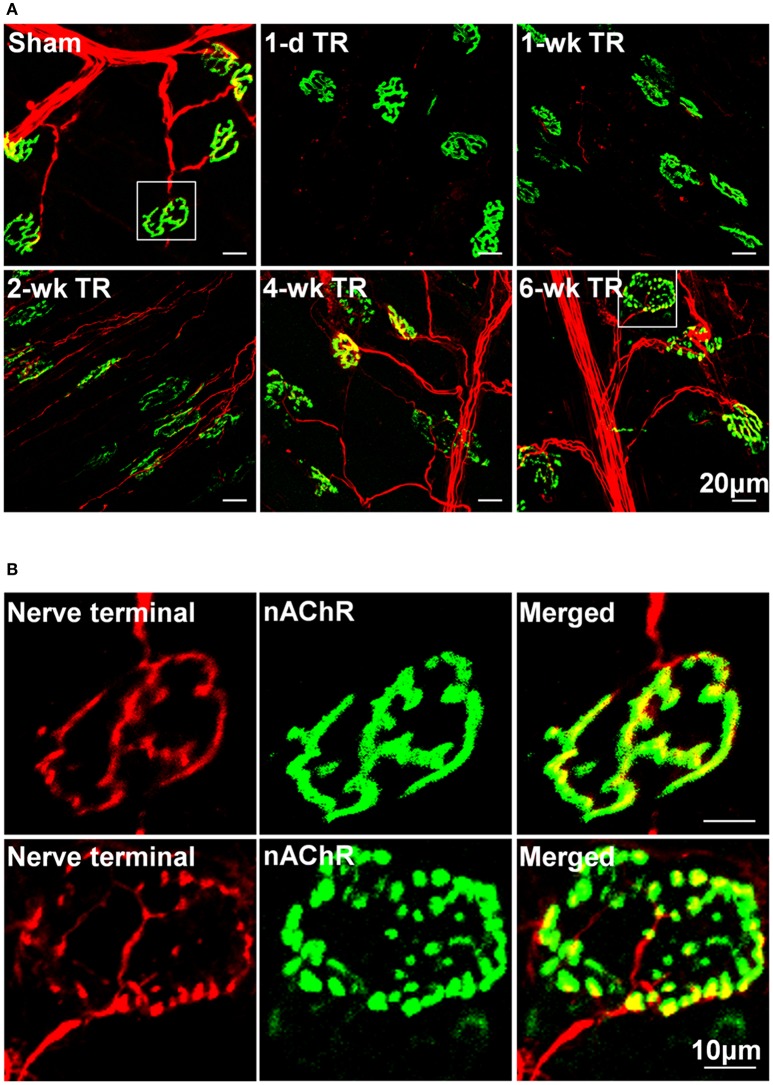
**(A)** Morphological changes in NMJs located on gastrocnemius muscles from sham mice and mice with different periods of tourniquet release after 3 h of tourniquet (1-d TR, 1-wk TR, 2-wk TR, 4-wk TR, and 6-wk TR). **(B)** Amplified photomicrographs for one NMJ in sham and 6-wk TR mice. Synaptophysin and neurofilament 200 (red color) and α-bungarotoxin (BTX; green color) were used to label presynaptic nerve terminals and post-synaptic nAChR clusters in NMJs.

Motor nerve terminals and nAChR clusters in NMJs were labeled by neurofilament and synaptophysin, and BTX, respectively. In NMJs, the structure of pretzel-like nAChR clusters normally remained intact, and motor nerve terminals innervated nAChR clusters to form synapses for signal transmission (Figures 1A,B). TR significantly induced alterations of NMJ morphology (Figure 1). In 1-d TR and 3-d TR, all motor nerve terminals were eliminated, but the pattern of nAChR clusters had non-significant changes. In 1-wk TR, some motor nerve fibers appeared and a few motor nerve terminals innervated nAChR clusters, whereas there were no significant alterations in nAChR clusters. In 2-wk TR, although many motor nerve fibers regenerated within muscle nerve fascicles and approached NMJs, these motor nerve fibers did not enter endplates or only a part of motor nerve terminals formed synapses with nAChR clusters. At the same time, many nAChR clusters were fragmented in 2-wk TR. In 4-wk TR, motor nerve terminals began to sprout into endplates and form new NMJs, and many nAChR clusters remained to be fragmented. In 6-wk TR, motor nerve terminals almost overlapped with nAChR clusters, whereas some nAChR clusters were still fragmented (Figure 1).

#### Quantification for alteration of motor nerve terminals

**Figure 2:**
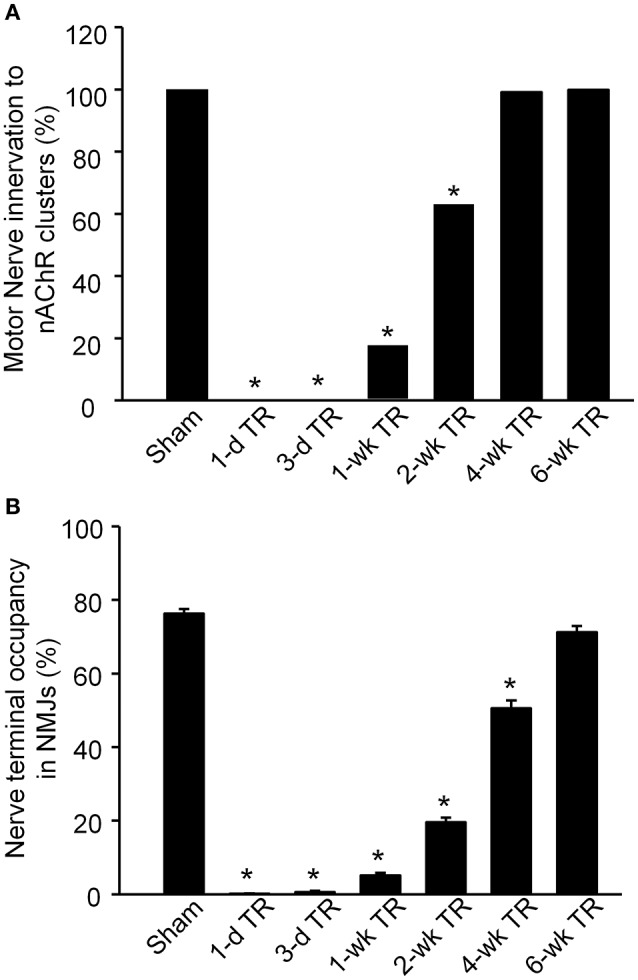
**Motor nerve innervation to nAChR clusters on gastrocnemius muscles from sham mice and mice with different periods of tourniquet release after 3 h of tourniquet (1-d TR, 3-d TR, 1-wk TR, 2-wk TR, 4-wk TR, and 6-wk TR)**. **(A)** Motor nerve innervation to nAChR clusters. **(B)** Nerve terminal occupancy in NMJs. Data are mean ± SE, *n* = 43–159 NMJs from 3 to 9 mice in each group. ^*^P < 0.05 vs. sham.

The alteration of motor nerve terminals in the NMJ was then quantified (Figure 2). In sham animals, all nAChR clusters were innervated by motor nerve terminals (100%, 159 NMJs from 9 mice). In 1-d TR and 3-d TR, motor nerve terminals in the gastrocnemius muscle wholly disappeared in all NMJs (1-d TR: 0%, 43 NMJs from 3 mice; 3-d TR: 0%, 64 NMJ from 4 mice; *p* < 0.05 vs. sham). In 1-wk TR, a few motor nerve terminals re-innervated nAChR clusters (17.2%, 128 NMJ from 5 mice, *p* < 0.05 vs. sham). From 2-wk TR, nAChR clusters were gradually re-innervated by motor nerve terminals, with longer TR time, even reaching normal levels in 6-wk TR (2-wk TR: 62.9%, 159 NMJ 159 NMJs from 5 mice; 4-w TR: 99.2%, 125 NMJ from 4 mice; 6-w TR: 100%, 100 NMJ from 4 mice; Figure 2A).

Although, motor nerve terminals innervate nAChR clusters in NMJs, the whole area of one nAChR cluster does not overlap with the innervated motor nerve terminal. Normally, the overlapping area is high in healthy NMJs (Tintignac et al., 2015). Nerve terminal occupancy in NMJs was calculated to compare the overlapping area in all experimental groups (Figure 2B). In sham mice, nerve terminal occupancy in NMJs was 76.31% ± 1.24. In 1-d TR and 3-d TR, there was no nerve terminal occupancy in NMJs (*p* < 0.05 vs. sham). From 1-wk TR to 6-wk TR, nerve terminal occupancy in NMJs was gradually restored with longer time of TR, especially approaching the normal level in 6-wk TR (5.14% ± 0.77 for 1-wk TR, 19.64% ± 1.27 for 2-wk TR, 50.58 % ± 2.17 for 4-wk TR, and 71.30% ± 1.65 for 6-wk TR; Figure 2B).

#### Quantification for nAChR clusters

**Figure 3:**
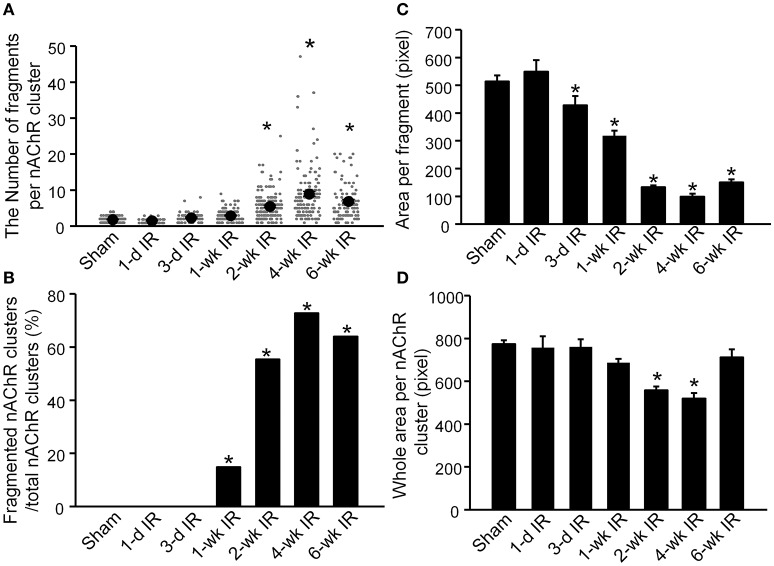
**Alterations of nAChR clusters on gastrocnemius muscles from sham mice and mice with different periods of tourniquet release after 3 h of tourniquet (1-d TR, 3-d TR, 1-wk TR, 2-wk TR, 4-wk TR, and 6-wk TR)**. **(A)** The number of fragments per nAChR cluster. **(B)** Fragmented nAChR clusters per total nAChR clusters. **(C)** Area per fragment. **(D)** Whole area per nAChR cluster. The number of discrete fragments per nAChR cluster ≥5 was considered as a fragmented nAChR cluster. Data are mean ± SE, *n* = 43–159 nAChR clusters from 3 to 9 mice in each group. ^*^P < 0.05 vs. sham.

Changes in nAChR clusters were also quantified into 4 variables (Figure 3). The number of fragments in each individual nAChR cluster was no more than 4 in sham animals (1.83 ± 0.07, 159 nAChR clusters from 9 mice, Figure 3A). Compared to sham mice, there were no significant changes in the number of fragments per nAChR cluster before 1-wk TR (1-d TR: 1.55 ± 0.10, 43 nAChR clusters from 3 mice; 3-d RR: 2.33 ± 0.17, 64 nAChR clusters from 4 mice; 1-wk TR: 2.92 ± 0.14, 128 nAChR clusters from 5 mice; *p* > 0.05 vs. sham). From 2-wk TR to 6-wk TR, the number of fragments per nAChR cluster was significantly increased (2-wk TR: 5.50 ± 0.30, 159 nAChR clusters from 5 mice; 4-wk TR: 8.94 ± 0.67, 125 nAChR clusters from 4 mice; 6-wk TR: 6.88 ± 0.50, 100 nAChR clusters from 4 mice; *p* < 0.05 vs. sham; Figure 3A).

When the number of discrete fragments per nAChR cluster ≥5 was considered as a fragmented nAChR cluster, the percentage of fragmented nAChR clusters began to increase from 1-wk TR, and lasted until 6-wk TR (0% for sham, 0% for 1-d TR, 0% for 3-d TR, 14.8% for 1-wk TR, 55.4% for 2-wk TR, 72.8% for 4-wk TR, and 64% for 6-wk TR; Figure 3B).

Another variable, the area per fragment, began to decrease from 3-d TR, and gradually reduced to a lower level with longer TR time (516.6 ± 21.6 for sham, 551.1 ± 42.3 for 1-d TR, 430.8 ± 33.7 for 3-d TR, 320.5 ± 6.5 for 1-wk TR, 135.1 ± 18.1 for 2-wk TR, 101.7 ± 10.9 for 4-wk TR, and 150.4 ± 10.7 for 6-wk TR; Figure 3C).

There were no changes in the area per nAChR cluster (the area per fragment times the number of fragments) before 1-wk TR (761.1 ± 53.8 for 1-d TR, 764.7 ± 35.1 for 3-d TR, and 689.4 ± 17.9 for 1-wk TR), compared to sham animals (777.34 ± 17.98, Figure 3D). The area per nAChR cluster was markedly reduced in 2-wk TR and 4-wk TR (561.6 ± 18 and 523.4 ± 25.4, *p* < 0.05 vs. sham), but recovered to the normal level in 6-wk TR (717.1 ± 37.1, Figure 3D).

### Functional changes of NMJs during TR

**Figure 4:**
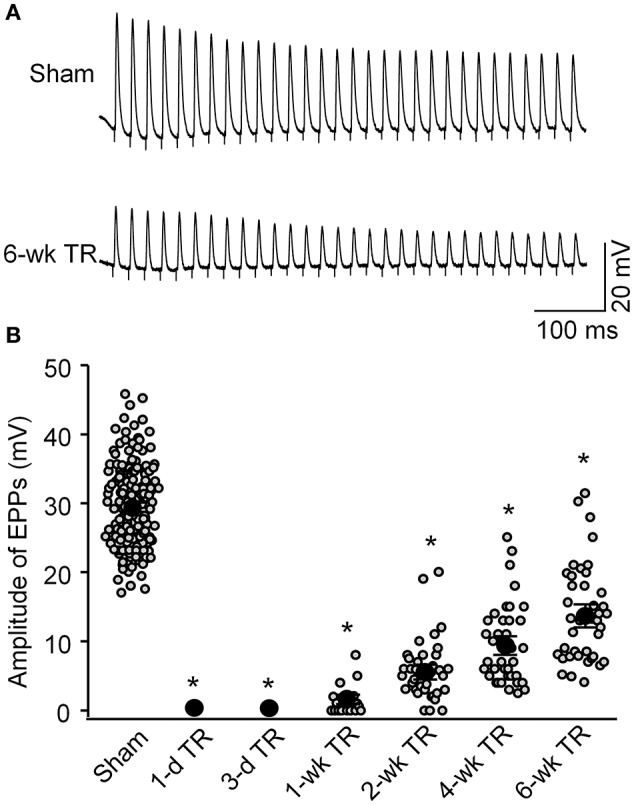
**Sciatic nerve-stimulated endplate potentials (EPPs) recorded in gastrocnemius muscles from sham mice and mice with different periods of tourniquet release after 3 h of tourniquet (1-d TR, 3-d TR, 1-wk TR, 2-wk TR, 4-wk TR, and 6-wk TR). (A)** Representative EPP recording in gastrocnemius muscles from sham and 6-wk TR mice. **(B)** Mean data illustrating the amplitude of EPPs recorded in gastrocnemius muscles from all experimental groups. Data are mean ± SE, *n* = 0–135 EPPs from 3 to 12 mice in each group. ^*^P < 0.05 vs. sham.

As noted, the NMJ normally conveys electrical signals from the motor nerve to the skeletal muscle and initiates skeletal muscle contraction. As the index of neuromuscular function, sciatic nerve-stimulated EPPs in the gastrocnemius muscle were recorded in all experimental groups (Figure 4). In the sham group, the amplitude of sciatic nerve-stimulated EPPs was 29.4 ± 0.6 mV (135 NMJs from 12 mice). In 1-d TR and 3-d TR, sciatic nerve-stimulated EPPs were not detectable. After 1-wk TR, the amplitude of sciatic nerve-stimulated EPPs gradually increased with longer TR time (1-wk TR: 1.5 ± 0.5 mV, 20 NMJs from 3 mice; 2-wk TR: 5.8 ± 0.7 mV, 38 NMJs from 3 mice; 4-wk TR: 9.5 ± 0.9 mV, 38 NMJs from 4 mice; 6-wk TR, 13.9 ± 1.1 mV, 40 NMJs from 3 mice; *p* < 0.05 vs. sham). Of more importance, the amplitude of sciatic nerve-stimulated EPPs did not recover to the normal level, even in 6-wk TR (Figure 4).

## Discussion

In the present study, we found that structural changes of motor nerve terminals and nAChR clusters in NMJs located on mouse gastrocnemius muscles had different timing patterns after TR. Motor nerve terminals completely disappeared in 1-d TR and 3-d TR, and then gradually regenerated with longer TR time, even re-innervating to all nAChR clusters in 6-wk TR. nAChR clusters had no significant alterations in 1-d TR and 3-d TR, but then over half of nAChR clusters were fragmented with longer TR time (from 2-wk TR to 6-wk TR). As the index of neuromuscular function, sciatic nerve-stimulated EPPs in the gastrocnemius muscle were not detectable in 1-d TR and 3-d TR. Although, the amplitude of sciatic nerve-stimulated EPPS gradually increased with longer TR time, amplitude did not recover to normal levels even in 6-wk TR. These findings indicate that the morphological damage of NMJs, including motor nerve terminals and nAChR clusters, causes neuromuscular dysfunction in mouse limb TR model.

When the limb is subjected to hypoxia-reperfusion in *ex-vivo* nerve and muscle preparations, motor nerves are the most vulnerable neuromuscular component (Baxter et al., 2008). In general, the extent of tissue damage depends on the duration of tourniquet application, and this has been found to be so in patients with tourniquet use and in the animal limb TR model. Most clinical studies support that no more than 60–90 min of tourniquet are the upper limit of safe tourniquet time in operative theaters and pre-hospital settings (Beekley et al., 2008; Doyle and Taillac, 2008). Thirty minutes of tourniquet application and subsequent release for 24 h only result in minor morphological changes of motor nerve terminals, whereas more than 30 min of tourniquet causes denervation of motor endplates in the skeletal muscle (David et al., 2007). Three hours of ischemia induced by artery occlusion completely block nerve conduction, and subsequent reperfusion disrupts the structure of motor nerves (Blunt, 1960; Makitie and Teravainen, 1977; Schmelzer et al., 1989). Tombol et al. also reported ultrastructural changes of the NMJ in a rat model of 2 h of tourniquet followed by 4 weeks of release (Tombol et al., 2002). These structural changes include loss of synaptic vesicles, disruption of the presynaptic membrane, mitochondria dysfunction, and development of vacuoles in the NMJ (Tombol et al., 2002). In particular, motor nerve degeneration is the most severe at 24 h of tourniquet release, and lasts about 4 weeks after tourniquet release (Tombol et al., 2002). In the present study, we found that 3 h of tourniquet application and subsequent release completely wiped out motor nerve terminals in 1-d TR and 3-d TR (Figures 1, 2). Additionally, the regeneration of motor nerve terminals began at 1-wk TR, and motor nerve innervation to nAChR clusters and motor nerve occupancy in NMJs gradually increased with longer TR time, even reaching normal levels in 6-wk TR (Figures 1, 2). These results are consistent with data from other two research groups (Tombol et al., 2002; Iida et al., 2003).

A high density of nAChRs is the hallmark of NMJs. Highly compacted nAChR clusters ensure skeletal muscle contraction in response to each nerve impulse in healthy skeletal muscles (Wood and Slater, 2001). In adult skeletal muscles, nAChRs at the NMJ are clustered and stabilized on the skeletal muscle at a half-life of about 14 days (Akaaboune et al., 1999). Even if muscle fibers are ablated, a low level of nAChRs persists for some time (Li and Thompson, 2011). Stabilization of nAChR clusters results from nAChR recycling, regulated by the motor nerve terminal and by skeletal muscle activity (Akaaboune et al., 1999; Yampolsky et al., 2010; Li and Thompson, 2011; Ouanounou et al., 2016). With motor nerve denervation and aging, nAChR clusters undergo marked morphological remodeling, with a high percentage of nAChR cluster fragmentation (Falk et al., 2015; Tintignac et al., 2015). In the present study, although morphological changes of nAChR clusters were not as rapid as motor nerve degeneration, the removal of nAChRs from the skeletal muscle membrane gradually increased the number of fragments per nAChR cluster and decreased the area per fragment in the nAChR cluster with longer TR time (Figure 3). While criteria such as “5 or more” fragments have been used to classify the nAChR cluster as either “fragmented” or “non-fragmented” nAChR clusters (Valdez et al., 2010; Falk et al., 2015; Willadt et al., 2016), our findings demonstrate that over half of nAChR clusters were still fragmented in 6-wk TR, although motor nerve innervation to nAChR clusters and motor nerve terminal occupancy in NMJs were recovered to normal levels.

For neuromuscular transmission of motor nerve impulses, ACh released from motor nerve terminals binds to nAChRs in the endplate membrane and depolarizes the skeletal muscle so as to fire action potentials and muscle contractions. The motor nerve terminal can spontaneously induce fusion of a few synaptic vesicles for Ach release without motor nerve impulses, and this induces a very small depolarization in the endplate membrane, called the mEPP. The excitation of motor nerves causes simultaneous fusion of dozens of vesicles, and these elicit a local depolarization of the endplate by ~30 mV, called the EPP. EPPs initiated with motor nerve excitation can be used to evaluate the efficiency of NMJ transmission (Tintignac et al., 2015; Zhang et al., 2017). In the present study, sciatic nerve-stimulated EPPs could not be recorded in 1-d TR and 3-d TR. Although the amplitude of sciatic nerve-stimulated EPPs gradually increased with longer TR time, it was not recovered to the normal level, even in 6-wk TR (Figure 4). Both morphological alterations of NMJs (motor nerve terminals and nAChR clusters) and resulting of sciatic nerve stimulated-EPPs during the TR suggest that motor nerve denervation could be mainly involved in disability of NMJ transmission during early stage of TR, while fragmentation of nAChR clusters possibly is the key factor that causes inefficiency of NMJ transmission during late-stage of TR. Some previous studies have shown that fragmentation of an individual nAChR cluster might correlate with a decline in efficacy of the neuromuscular transmission in pathophysiological states (Maselli et al., 1995; Gonzalez-Freire et al., 2014; Steinbeck et al., 2015; Vescovo, 2015; Willadt et al., 2016). Our current study further demonstrates that dysfunction of NMJ transmission is mediated by both motor nerve denervation and the fragmentation of nAChR clusters during TR.

We clearly recognize that it is optimal to simultaneously measure the morphology (the structure of the NMJ) and function (EPPs) in the same single NMJ. However, this is currently not feasible, because it requires advanced techniques not yet developed. Nevertheless, by comparing the structure of the NMJ and sciatic nerve-stimulated EPPs at different periods of tourniquet release after 3 h of tourniquet application, the current study confirmed the correlation between morphological changes of the NMJ and dysfunction of NMJ transmission.

Although, motor nerve innervation and occupancy in NMJs were recovered to normal levels at 6-wk TR, it is possible that the functional alteration of motor nerves also affects the amplitude of sciatic nerve-stimulated EPPs. In future study, therefore, we will measure conduction velocity of motor nerves and acetylcholine release from motor nerve terminals in the gastrocnemius muscle, using the electrophysiological method (Oh et al., 2010) and micro-dialysis/HPLC technique (Huang et al., 2007), which can verify or exclude all of these possibilities.

In conclusion, TR resulted in acute and long-term morphological and functional alterations of NMJs in mouse gastrocnemius muscles. Motor nerve denervation mainly mediated disability of NMJ transmission during early stage of TR, whereas fragmentation of nAChR clusters could dampen NMJ transmission during late-stage of TR. The results suggest that, for tourniquet-induced extremity injuries, the animal model presented in this study is very useful to explore the potential cellular and molecular mechanisms of tourniquet-induced injuries, including NMJ dysfunction. Thereby, there is high potential to optimize the tourniquet method and discover therapeutic interventions that will improve outcomes for patients with tourniquet-induced injuries.

## Author contributions

HT, DZ, RC, YL designed and performed experiments, analyzed data, and prepared the manuscript. HT, RM, MW, YL wrote, edited, and revised manuscript. HT, DZ, RC, RM, MW, YL approved the version to be published. HT, DZ, RC, RM, MW, YL agreed to be accountable for all aspects of the work.

### Conflict of interest statement

The authors declare that the research was conducted in the absence of any commercial or financial relationships that could be construed as a potential conflict of interest.

## References

[B1] Akaaboune M. Culican S. M. Turney S. G. Lichtman J. W. (1999). Rapid and reversible effects of activity on acetylcholine receptor density at the neuromuscular junction *in vivo*. Science 286, 503–507. 10521340 10.1126/science.286.5439.503

[B2] Baxter B. Gillingwater T. H. Parson S. H. (2008). Rapid loss of motor nerve terminals following hypoxia-reperfusion injury occurs via mechanisms distinct from classic Wallerian degeneration. J. Anat. 212, 827–835. 10.1111/j.1469-7580.2008.00909.x 18510509 PMC2423403

[B3] Beekley A. C. Sebesta J. A. Blackbourne L. H. Herbert G. S. Kauvar D. S. Baer D. G.. (2008). Prehospital tourniquet use in Operation Iraqi Freedom: effect on hemorrhage control and outcomes. J. Trauma 64, S28–S37. 10.1097/ta.0b013e318160937e 18376169

[B4] Blunt M. J. (1960). Ischemic degeneration of nerve fibers. Arch. Neurol. 2, 528–536. 10.1001/archneur.1960.03840110042005 13801759

[B5] Creager M. A. Kaufman J. A. Conte M. S. (2012). Clinical practice. Acute limb ischemia. N. Engl. J. Med. 366, 2198–2206. 10.1056/NEJMcp1006054 22670905

[B6] Darabid H. Perez-Gonzalez A. P. Robitaille R. (2014). Neuromuscular synaptogenesis: coordinating partners with multiple functions. Nat. Rev. Neurosci. 15, 703–718. 10.1038/nrn3821 25493308

[B7] David G. Nguyen K. Barrett E. F. (2007). Early vulnerability to ischemia/reperfusion injury in motor terminals innervating fast muscles of SOD1-G93A mice. Exp. Neurol. 204, 411–420. 10.1016/j.expneurol.2006.12.021 17292357 PMC2097955

[B8] Domingues-Faria C. Vasson M. P. Goncalves-Mendes N. Boirie Y. Walrand S. (2016). Skeletal muscle regeneration and impact of aging and nutrition. Ageing Res. Rev. 26, 22–36. 10.1016/j.arr.2015.12.004 26690801

[B9] Doyle G. S. Taillac P. P. (2008). Tourniquets: a review of current use with proposals for expanded prehospital use. Prehosp. Emerg. Care 12, 241–256. 10.1080/10903120801907570 18379924

[B10] Falk D. J. Todd A. G. Lee S. Soustek M. S. ElMallah M. K. Fuller D. D.. (2015). Peripheral nerve and neuromuscular junction pathology in Pompe disease. Hum. Mol. Genet. 24, 625–636. 10.1093/hmg/ddu476 25217571 PMC4291243

[B11] Froehner S. C. Luetje C. W. Scotland P. B. Patrick J. (1990). The postsynaptic 43K protein clusters muscle nicotinic acetylcholine receptors in Xenopus oocytes. Neuron 5, 403–410. 10.1016/0896-6273(90)90079-u 1698395

[B12] Gonzalez-Freire M. de Cabo R. Studenski S. A. Ferrucci L. (2014). The neuromuscular junction: aging at the crossroad between nerves and muscle. Front. Aging Neurosci. 6:208. 10.3389/fnagi.2014.00208 25157231 PMC4127816

[B13] Grumbles R. M. Almeida V. W. Casella G. T. Wood P. M. Hemstapat K. Thomas C. K. (2012). Motoneuron replacement for reinnervation of skeletal muscle in adult rats. J. Neuropathol. Exp. Neurol. 71, 921–930. 10.1097/NEN.0b013e31826cf69a 22964786 PMC3760019

[B14] Huang Y. T. Cheng C. J. Lai T. F. Tsai T. R. Tsai T. H. Chuo W. H.. (2007). An investigation of acetylcholine released in skeletal muscle and protein unbound drug released in blood based on the pyridostigmine bromide (pretreatment drug) sustained-release pellets by microdialysis technique in the rabbit model. Neurosci. Lett. 416, 302–306. 10.1016/j.neulet.2007.02.019 17336457

[B15] Huh K. H. Fuhrer C. (2002). Clustering of nicotinic acetylcholine receptors: from the neuromuscular junction to interneuronal synapses. Mol. Neurobiol. 25, 79–112. 10.1385/MN:25:1:079 11890459

[B16] Iida H. Schmelzer J. D. Schmeichel A. M. Wang Y. Low P. A. (2003). Peripheral nerve ischemia: reperfusion injury and fiber regeneration. Exp. Neurol. 184, 997–1002. 10.1016/s0014-4886(03)00385-6 14769393

[B17] Ijkema-Paassen J. Meek M. F. Gramsbergen A. (2002). Reinnervation of muscles after transection of the sciatic nerve in adult rats. Muscle Nerve 25, 891–897. 10.1002/mus.10130 12115979

[B18] Inaba K. Siboni S. Resnick S. Zhu J. Wong M. D. Haltmeier T.. (2015). Tourniquet use for civilian extremity trauma. J. Trauma Acute Care Surg. 79, 232–237. 10.1097/TA.0000000000000747 26218691

[B19] Kam P. C. A. Kavanaugh R. Yoong F. F. Y. (2001). The arterial tourniquet: pathophysiological consequences and anaesthetic implications. Anaesthesia 56, 534–545. 10.1046/j.1365-2044.2001.01982.x 11412159

[B20] Kang H. Lichtman J. W. (2013). Motor axon regeneration and muscle reinnervation in young adult and aged animals. J. Neurosci. 33, 19480–19491. 10.1523/JNEUROSCI.4067-13.2013 24336714 PMC6618761

[B21] Li Y. Thompson W. J. (2011). Nerve terminal growth remodels neuromuscular synapses in mice following regeneration of the postsynaptic muscle fiber. J. Neurosci. 31, 13191–13203. 10.1523/JNEUROSCI.2953-11.2011 21917802 PMC3181159

[B22] Makitie J. Teravainen H. (1977). Peripheral nerve injury and recovery after temporary ischemia. Acta Neuropathol. 37, 55–63. 842295 10.1007/BF00684541

[B23] Maselli R. A. Wollmann R. Roos R. (1995). Function and ultrastructure of the neuromuscular junction in post-polio syndrome. Ann. N.Y. Acad. Sci. 753, 129–137. 10.1111/j.1749-6632.1995.tb27539.x 7611622

[B24] McMahan U. J. Edgington D. R. Kuffler D. P. (1980). Factors that influence regeneration of the neuromuscular junction. J. Exp. Biol. 89, 31–42. 7009777 10.1242/jeb.89.1.31

[B25] Nishizawa T. Tamaki H. Kasuga N. Takekura H. (2003). Degeneration and regeneration of neuromuscular junction architecture in rat skeletal muscle fibers damaged by bupivacaine hydrochloride. J. Muscle Res. Cell Motil. 24, 527–537. 10.1023/B:JURE.0000009905.89618.33 14870968

[B26] Ochoa J. Danta G. Fowler T. J. Gilliatt R. W. (1971). Nature of the nerve lesion caused by a pneumatic tourniquet. Nature 233, 265–266. 4999642 10.1038/233265a0

[B27] Oh S. S. Hayes J. M. Sims-Robinson C. Sullivan K. A. Feldman E. L. (2010). The effects of anesthesia on measures of nerve conduction velocity in male C57Bl6/J mice. Neurosci. Lett. 483, 127–131. 10.1016/j.neulet.2010.07.076 20691755 PMC2941214

[B28] Ouanounou G. Baux G. Bal T. (2016). A novel synaptic plasticity rule explains homeostasis of neuromuscular transmission. Elife 5:e12190. 10.7554/eLife.12190 27138195 PMC4854514

[B29] Phillips W. D. Kopta C. Blount P. Gardner P. D. Steinbach J. H. Merlie J. P. (1991). ACh receptor-rich membrane domains organized in fibroblasts by recombinant 43-kildalton protein. Science 251, 568–570. 10.1126/science.1703661 1703661

[B30] Sanes J. R. Lichtman J. W. (2001). Induction, assembly, maturation and maintenance of a postsynaptic apparatus. Nat. Rev. Neurosci. 2, 791–805. 10.1038/35097557 11715056

[B31] Scerbo M. H. Mumm J. P. Gates K. Love J. D. Wade C. E. Holcomb J. B.. (2016). Safety and appropriateness of tourniquets in 105 civilians. Prehosp. Emerg. Care 20, 712–722. 10.1080/10903127.2016.1182606 27245978 PMC5104170

[B32] Scheib J. Hoke A. (2013). Advances in peripheral nerve regeneration. Nat. Rev. Neurol. 9, 668–676. 10.1038/nrneurol.2013.227 24217518

[B33] Schmelzer J. D. Zochodne D. W. Low P. A. (1989). Ischemic and reperfusion injury of rat peripheral nerve. Proc. Natl. Acad. Sci. U.S.A. 86, 1639–1642. 10.1073/pnas.86.5.1639 2922402 PMC286754

[B34] Slater C. R. (1982). Neural influence on the postnatal changes in acetylcholine receptor distribution at nerve-muscle junctions in the mouse. Dev. Biol. 94, 23–30. 10.1016/0012-1606(82)90064-1 7152105

[B35] Slater C. R. Allen E. G. (1985). Acetylcholine receptor distribution on regenerating mammalian muscle fibers at sites of mature and developing nerve-muscle junctions. J. Physiol. 80, 238–246. 3834077

[B36] Steinbeck L. Ebner N. Valentova M. Bekfani T. Elsner S. Dahinden P.. (2015). Detection of muscle wasting in patients with chronic heart failure using C-terminal agrin fragment: results from the Studies Investigating Co-morbidities Aggravating Heart Failure (SICA-HF). Eur. J. Heart Fail. 17, 1283–1293. 10.1002/ejhf.400 26449626

[B37] Tintignac L. A. Brenner H. R. Ruegg M. A. (2015). Mechanisms regulating neuromuscular junction development and function and causes of muscle wasting. Physiol. Rev. 95, 809–852. 10.1152/physrev.00033.2014 26109340

[B38] Tombol T. Pataki G. Nemeth A. Hamar J. (2002). Ultrastructural changes of the neuromuscular junction in reperfusion injury. Cells Tissues Organs 170, 139–150. 11731702 10.1159/000046187

[B39] Tran T. P. Tu H. Liu J. Muelleman R. L. Li Y.-L. (2012). Mitochondria-derived superoxide links to tourniquet-induced apoptosis in mouse skeletal muscle. PLoS ONE 7:e43410. 10.1371/journal.pone.0043410 22912870 PMC3422247

[B40] Tran T. P. Tu H. Pipinos I. I. Muelleman R. L. Albadawi H. Li Y. L. (2011). Tourniquet-induced acute ischemia-reperfusion injury in mouse skeletal muscles: involvement of superoxide. Eur. J. Pharmacol. 650, 328–334. 10.1016/j.ejphar.2010.10.037 21036124 PMC3008320

[B41] Valdez G. Tapia J. C. Kang H. Clemenson G. D. Jr. Gage F. H. Lichtman J. W.. (2010). Attenuation of age-related changes in mouse neuromuscular synapses by caloric restriction and exercise. Proc. Natl. Acad. Sci. U.S.A. 107, 14863–14868. 10.1073/pnas.1002220107 20679195 PMC2930485

[B42] Vescovo G. (2015). Neuromuscular junction fragmentation and muscle wasting in heart failure: a sharp cut from a Sica sword? Eur. J. Heart Fail. 17, 1216–1218. 10.1002/ejhf.437 26647214

[B43] Willadt S. Nash M. Slater C. R. (2016). Age-related fragmentation of the motor endplate is not associated with impaired neuromuscular transmission in the mouse diaphragm. Sci. Rep. 6:24849. 10.1038/srep24849 27094316 PMC4837408

[B44] Wood S. J. Slater C. R. (2001). Safety factor at the neuromuscular junction. Prog. Neurobiol. 64, 393–429. 10.1016/s0301-0082(00)00055-1 11275359

[B45] Yampolsky P. Pacifici P. G. Lomb L. Giese G. Rudolf R. Roder I. V.. (2010). Time lapse *in vivo* visualization of developmental stabilization of synaptic receptors at neuromuscular junctions. J. Biol. Chem. 285, 34589–34596. 10.1074/jbc.M110.168880 20813841 PMC2966074

[B46] Yu X. M. Hall Z. W. (1994). The role of the cytoplasmic domains of individual subunits of the acetylcholine receptor in 43 kDa protein-induced clustering in COS cells. J. Neurosci. 14, 785–795. 8301361 10.1523/JNEUROSCI.14-02-00785.1994 PMC6576804

[B47] Zhang D. Wang D. Pipinos I. I. Muelleman R. L. Li Y. L. (2017). Dexamethasone promotes long-term functional recovery of neuromuscular junction in a murine model of tourniquet-induced ischaemia-reperfusion. Acta Physiol. 219, 453–464. 10.1111/apha.12737 27306588

[B48] Zietlow J. M. Zietlow S. P. Morris D. S. Berns K. S. Jenkins D. H. (2015). Prehospital use of hemostatic bandages and tourniquets: translation from military experience to implementation in civilian trauma care. J. Spec. Oper. Med. 15, 48–53. 26125164 10.55460/1P70-3H9D

